# The neural implausibility of the diffusion decision model doesn’t matter for cognitive psychometrics, but the Ornstein-Uhlenbeck model is better

**DOI:** 10.3758/s13423-024-02520-5

**Published:** 2024-05-14

**Authors:** Jia-Shun Wang, Christopher Donkin

**Affiliations:** 1 Department of Psychology, Ludwig-Maximilians-Universität München, Munich, Germany; 2 Graduate School of Systemic Neurosciences, Ludwig-Maximilians-Universität München, Munich, Germany

**Keywords:** Response time models, Decision making, Mathematical modelling, Model mimicry, Cognitive psychometrics

## Abstract

In cognitive psychometrics, the parameters of cognitive models are used as measurements of the processes underlying observed behavior. In decision making, the diffusion decision model (DDM) is by far the most commonly used cognitive psychometric tool. One concern when using this model is that more recent theoretical accounts of decision-making place more emphasis on neural plausibility, and thus incorporate many assumptions not found in the DDM. One such model is the Ising Decision Maker (IDM), which builds from the assumption that two pools of neurons with self-excitation and mutual inhibition receive perceptual input from external excitatory fields. In this study, we investigate whether the lack of such mechanisms in the DDM compromises its ability to measure the processes it does purport to measure. We cross-fit the DDM and IDM, and find that the conclusions of DDM would be mostly consistent with those from an analysis using a more neurally plausible model. We also show that the Ornstein-Uhlenbeck Model (OUM) model, a variant of the DDM that includes the potential for leakage (or self-excitation), reaches similar conclusions to the DDM regarding the assumptions they share, while also sharing an interpretation with the IDM in terms of self-excitation (but not leakage). Since the OUM is relatively easy to fit to data, while being able to capture more neurally plausible mechanisms, we propose that it be considered an alternative cognitive psychometric tool to the DDM.

**Supplementary Information:**

The online version contains supplementary material available at 10.3758/s13423-024-02520-5.

## Introduction

In the field of cognition, the study of decision-making processes has been a topic of great importance. For relatively fast decisions during simple tasks, we typically use reaction time (RT) and accuracy (the probability of making a correct decision as measures of performance). Most successful models of such choice RTs and accuracy rates fall into the framework of evidence accumulation.

Many of the models within the evidence accumulation framework are variations of the diffusion decision model (DDM; Ratcliff, 1978; Ratcliff et al., 2016; Ratcliff & Rouder, 1998). The DDM explains the decision-making process as one of accumulating evidence over time until a decision threshold is reached and a response is made. Over the years, DDMs have been used to understand choices made across a range of paradigms, tasks, and conditions (Ratcliff, 2006; Ratcliff et al., 2010; Ratcliff & McKoon, 2008; Ratcliff & Smith, 2004).

One of the most common uses of the DDM is that called *Cognitive Psychometrics*, which refers to using cognitive process models as measurement tools to analyze the cognitive processes underlying behavior (Batchelder, 1998). The parameters of most cognitive models are assumed to have some meaning or interpretation, and in cognitive psychometrics they are used as measures of such things, replacing the more standard indicators of performance like mean accuracy or mean RT. The DDM has seen extensive use as a cognitive psychometric tool because it has parameters with clear interpretations. For example, in perceptual discrimination tasks, the drift rate has been used to measure differences in sensitivity to visual stimuli among individuals (Batchelder, 1998). In tasks involving speed-accuracy manipulation, the decision threshold parameter has been employed to study the caution in the decision-making process (Ratcliff & Rouder, 1998). The DDM has also been applied in studies on aging and clinical populations, helping to identify cognitive changes or impairments in decision-making (Ratcliff et al., 2003; Wiecki et al., 2015). It is worth noting here that one of the main reasons for the widespread use of the DDM is that it is relatively easy to fit to behavioral data (a requirement for getting parameter estimates).

As the years have progressed, more complex variants of the basic DDM have been proposed, such as the Racing Diffusion Model, Urgency-Gating Models, DDM with collapsing boundaries, and models that also capture confidence responses (Bogacz et al., 2006; Cisek et al., 2009; Ditterich, 2006, 2006; Drugowitsch et al., 2012; Ratcliff et al., 2016; Tillman et al., 2020; Van Den Berg et al., 2016). Many of these more complex models were introduced to account for patterns of choices and response times that are reliably seen in experiments. For example, Ratcliff and Rouder (1998) proposed that the start-point of evidence accumulation changes from trial-to-trial to explain the observation that incorrect responses are faster than correct responses when trying to answer quickly, and vice versa when trying to be accurate.

Our focus here is on another major reason for making decision-making models more complex, which is to introduce assumptions that are consistent with the neural and biological processes that are thought to underlie decision making. It can make sense to include such assumptions, since a complete explanation of decision making should be consistent with what we have learned from other decision-making research, even though it is sometimes unclear whether such processes have implications for the behavioral data observed in decision-making experiments. Some of the earliest decision models to focus on being neurally plausible are the spiking neural network model (Wong & Wang, 2006) and the Leaky Competing accumulator model (Usher & McClelland, 2001). While the spiking neural network model was built on the basis of realistic neural circuits, the Leaky Competing Accumulator model adds neurally plausible assumptions, like evidence decay and mutual inhibition, to a typical evidence-accumulation model framework.

In what follows we will explore the Ising Decision Maker (IDM), which is a more recently proposed neurobiologically inspired model of decision-making that draws from principles in statistical physics (Verdonck & Tuerlinckx, 2014). Verdonck and Tuerlinckx (2014) show that a biologically plausible decision process, built on self-excitative and mutually inhibitive neural populations, can be represented as a system state in a decision field, with responses being triggered when that state crosses into one of the available detection boxes. Broadly speaking, the decision process involves minimizing energy until the state of the decision system corresponds to one of the final choices. These authors approximate such a system within a more typical diffusion-like process, and show how it is related to the neurally plausible models mentioned earlier (i.e., the models in Usher & McClelland, 2001, and Wong & Wang, 2006).

From a Cognitive Psychomterics perspective, there are two downsides to making our models more complex. The first is that the models can become very computationally costly, and so cannot be easily fit to data using convenient optimization algorithms (e.g., Wong & Wang, 2006). The second issue is that in any given Cognitive Psychometrics analysis, the data will often not contain the kind of information that is necessary to constrain the estimation of the parameters of the model (Umakantha et al., 2022). This is especially true for something like neural plausibility, where the reasons for certain assumptions (e.g., the physical properties of neurons) may have only subtle and indirect consequences on the dependent variables that will be used to estimate the model parameters (e.g., response times and choices). These issues make such models inappropriate for Cognitive Psychometric applications, where we care about being able to estimate parameters robustly and with relative ease.

Having to rely on easy-to-use and simple models for Cognitive Psychometrics raises a potential problem, since models that can explain the relevant neural and biological data are likely more accurate reflections of the decision-making process than those that ignore such factors. In other words, in an ideal world, our Cognitive Psychometric models would be equivalent to our best-known explanations of all the relevant phenomena (i.e., behavioral data patterns, neural plausibility, etc.). With sufficient data to constrain the parameters of our most complete models, we could be confident that we were drawing the best available inferences from a model-based analysis.

As it is, we are faced with a tradeoff, since more neurally plausible assumptions in decision-making models will presumably make them more accurate but will often render such models useless as Cognitive Psychometric tools. This problem is a special case of the bias-variance tradeoff, and it leaves us preferring relatively simple models like the DDM when the goal is to estimate the parameters of our cognitive models (van Ravenzwaaij et al., 2017). However, if we are going to settle for using the simpler models, we should worry whether we might make substantially different conclusions than had we used more complex models.

The aim of this paper was to address this issue by providing a systematic comparison between the IDM and the DDM. We seek to determine whether the increased neural plausibility in the IDM results can lead to significantly different conclusions about decision processes compared to the simplest DDM, which is just an unbiased Wiener diffusion model. To strike a balance between biological plausibility and practicality, we also consider the Ornstein-Uhlenbeck Model (OUM) as a compromise (Busemeyer & Townsend, 1993). The OUM incorporates some neurally inspired assumptions while remaining applicable to data and readily usable for cognitive psychometrics. Through this investigation, we aim to shed light on the trade-offs between model complexity, biological plausibility, and the capacity to explain decision-making behaviors.

## Methods

We use the cross-fitting method using in Matzke and Wagenmakers (2009; see also Donkin et al., 2011) to investigate whether there is one-to-one correspondence between the core parameters of IDM and DDM and between that of IDM and OUM. Specifically, we will first simulate RT and accuracy data from one model, while allowing its core parameters to change freely, and fit those data with the other model, observing how the parameters of interest capture those manipulations. If the change of one core parameter in one model only has effect on the analogous parameter in the other model and not on the non-analogous parameters, we can conclude that there is convergent validity between the corresponding parameters in each model. However, if changes in one parameter are reflected in multiple parameters, then we may have caused to question the validity of certain analyses. The code is made available and can be accessed via https://github.com/Jiashun97/IDM.

### The outline of the models

#### Diffusion model

**Fig. 1:**
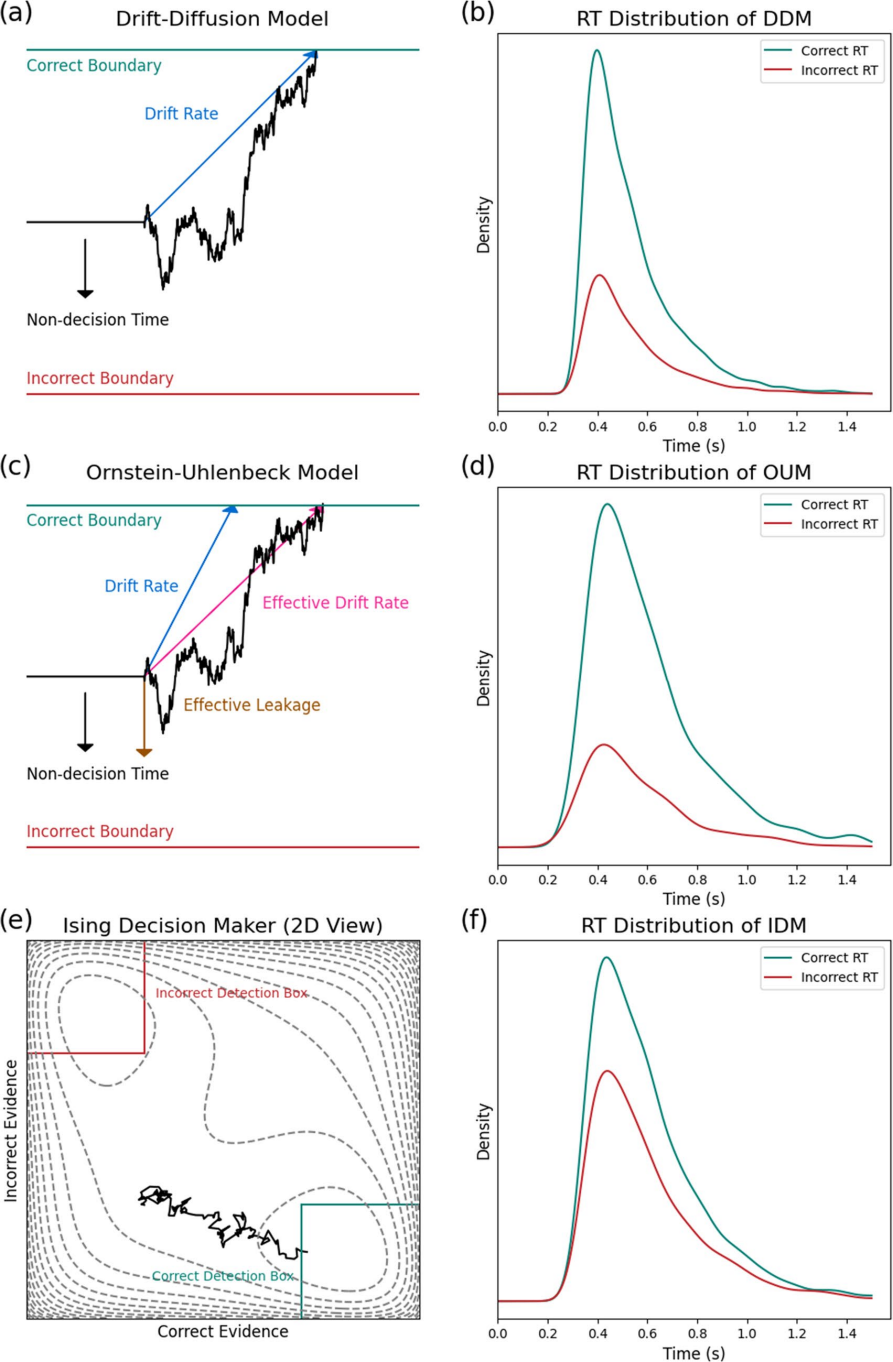
Illustrations of one decision trial in each of the three decision-making models we consider. (**a**) Drift-Diffusion Model (DDM): The wiggly line shows the evidence accumulation process. The average rate of evidence accumulation is the drift rate (blue). A correct decision is made when the system reaches the boundary of the correct option (green), and an incorrect decision is made when it reaches the boundary of the incorrect option (red). (**b**) The simulated reaction time (RT) distribution of DDM for correct (green) and incorrect responses (red). The smooth curve is obtained by applying a kernel density estimation method to data simulated from the model. We used the following parameter values: *v = 1, a = 0.5, ter = 0.3*. (**c**) Ornstein-Uhlenbeck Model (OUM): All three parameters are the same as those in DDM, but there is another parameter called effective leakage (or self-excitation). Unlike the constant drift rate in DDM, evidence accumulation in the OUM changes according to the state of the evidence accumulation. The drift rate (*v*) and the effective leakage (*k*) are combined into the effective drift rate (*v-kx*), which also depends on the evidence accumulated at that time step. The arrow (in brown) showing of the effect of effective leakage in the figure only serves for illustration purpose. (**d**) The simulated RT distribution of OUM for correct (green) and incorrect responses (red). We used the following parameter values: *v = 1, a = 0.5, Ter = 0.3, k = 3*. The difference between panels (**b**) and (**d**) is due to the introduction of effective leakage in the OUM. (**e**) Ising Decision Maker (IDM): Unlike DDM or OUM, the IDM decision variable has two dimensions. A decision is made when the system crosses one of the detection boxes (green for correct and red for incorrect). Grey dotted lines indicate the contours of free energy of the system. The system tends to stay in an area where the free energy is low. There are two local minima in the figure: the upper left one and the lower right one. The lower right one is deeper because it stands for the correct choice. (**f**) The simulated RT distribution of IDM for correct (green) and incorrect responses (red). The parameters of the IDM were chosen to generate data like the DDM, based on the results of our cross-fitting analysis reported below (*C = 0.1658, h = 0.5951, Ter = 0.2648, D = 0.0111*, *W*^*+*^ and *W*^*-*^ fixed to 52500 and 8400, respectively)

We are using the simple diffusion decision model in this study, shown in Fig. 1a, which assumes a single evidence accumulator that diffuses with a constant drift rate and Gaussian noise, eventually reaching one of the two boundaries corresponding to the two possible choices in the two-alternative forced-choice task. The model has an unbiased start point and assumes that none of its parameters varies across trials, unlike more complete DDM models (i.e., Ratcliff, 1978; Ratcliff & Rouder, 1998; Ratcliff & Tuerlinckx, 2002). This simple DDM has three core parameters: drift rate (v), boundary separation (a), and non-decision time (*T*_*er*_). It can be represented by this stochastic differential equation: dy = v*dt + c*dW, in which *y* is the current value of evidence accumulation, *c* is the diffusion constant and *W* is a Wiener process (i.e., the addition of Gaussian noise). The process begins at 0.5*a and terminates when *y* reaches either *a* or *0*, and the RT from the model is obtained by adding the non-decision time *T*_*er*_ to this first-passage time (see Fig. 1b for an example of the predicted RT distributions from the model). The evidence obtained by the DDM accumulator at each time step is proportional to the log likelihood ratio of the evidence, which corresponds to the drift rate. It can be thought of as an optimal decision maker because it implements the Neyman-Pearson procedure for the free-response paradigm (Bogacz et al., 2006).

#### Ornstein-Uhlenbeck Model

The Ornstein–Uhlenbeck Model (OUM), shown in Fig. 1c, is a simple variation of the DDM that adds an effective leakage parameter *k*, such that the rate of evidence change is not constant, but depends on the current state of evidence. The OUM has the form dy = (v-k*y)*dt + c*dW. Depending on the sign of effective leakage, *y* can accelerate or decelerate toward the response boundary. This parameter is thought to be related to reward and punishment in decision making (Busemeyer & Townsend, 1993). In all other ways, the OUM is the same as the DDM (see Fig. 1d for an example of the predicted RT distributions from the OUM).

#### Ising Decision Maker

The Ising Decision Maker (IDM) assumes two pools of neurons with self-excitation and mutual inhibition, both receiving external inputs from the stimulus being evaluated (Verdonck & Tuerlinckx, 2014). Before we go into the model and its parameters in more detail, it is worth noting that the IDM has three parameters that have direct analogues in the DDM: stimulus distinctness (*C*), detection box size (*h)*, and non-decision time (*T*_*er*_).

##### **Model architecture**

The IDM model consists of two neural pools and each pool has 1,000 neurons. One pool of neurons is responsible for evidence accumulation for the first response option and the other pool for the second response. Each pool receives external inputs (*B*_*1*_ and *B*_*2*_) from the task stimulus, has a self-excitation connection to itself, and receives inhibitory connection from the other pool.

The dynamics of the activation of the neurons in the IDM can be described as a decision state (e.g., a position in a two-dimensional space shown in Fig. 1e) that acts to minimize a third dimension (free energy), where the gradient of motion through that space is defined by a free-energy surface (i.e., the dotted contour lines in Fig. 1e). A decision is made once the decision state enters a certain region, shown as detection boxes in Fig. 1e, reflecting sufficient activation of one of the two pools of neurons.

##### **Model input**

To make the parameterization of the IDM as similar to that of the DDM, we can reformulate the two external fields (*B*_*1*_, *B*_*2*_) as follows: *B*_*1*_* = B*_*s*_*(1+C) + B*_*ns*_*,* and* B*_*2*_* = B*_*s*_*(1-C) + B*_*ns*_*.* Then we have stimulus distinctiveness (*C*), selective stimulus strength (*B*_*s*_), and non-selective stimulus strength (*B*_*ns*_). The stimulus distinctiveness is similar to the drift rate in DDM, which indicates stimulus quality. For instance, when *C = 0*, indicating an uninformative stimulus, then *B*_*1*_* = B*_*2*_* = B*_*s*_* + B*_*ns*_*.* When the stimulus is absolutely informative* (C = 1),* we have *B*_*1*_* = 2B*_*s*_* + B*_*ns*_*,* and* B*_*2*_* = B*_*ns*_*.* The stimulus distinctiveness (*C*) can also go negative, but we only consider the situation where *C > 0* since the process is symmetric.

##### Starting position

 The model needs to be initialized into a particular low free-energy state. Here, we always start the model at the position (0.2,0.2) since here the energy is low when the stimulus has not yet been introduced (see Fig. 2 in Verdonck & Tuerlinckx, 2014). The value of 0.2 indicates the proportion of correct (and incorrect) evidence that has been collected thus far, related to the proportion of neurons activated in the underlying network. The choice of value is based on previous applications of the model (Verdonck & Tuerlinckx, 2014). After stimulus onset, the external fields are activated and the free-energy surface changes shape. At this point, the decision process begins and the decision state moves to seek out a newly defined low free-energy state.

**Fig. 2:**
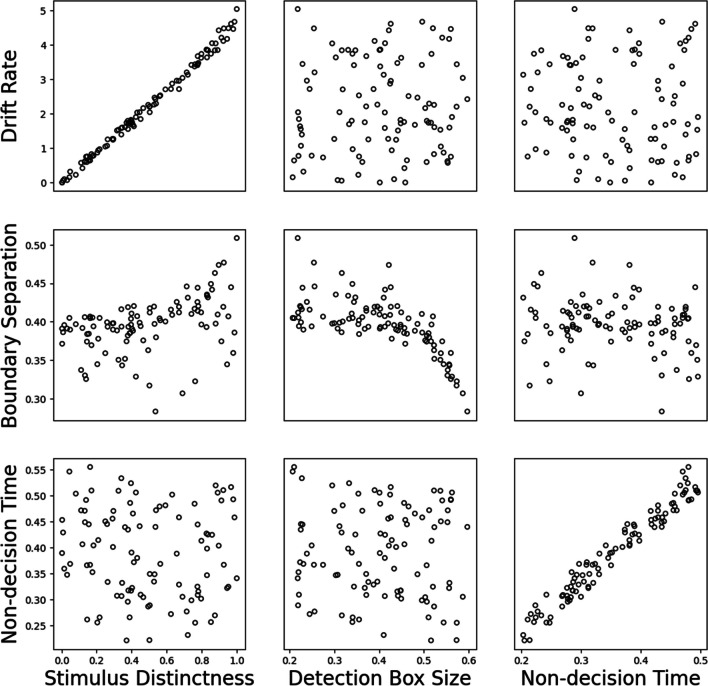
Results of fitting the Drift-Decision Model (DDM) to Ising Decision Maker (IDM)  data. The horizontal axes show the core parameters in IDM and the  vertical axes shows the analogous parameters in DDM. Subplots in the diagonal show the relationship  between the corresponding parameters. Off-diagonal subplots show the relationship between other noncorresponding parameters

##### **Macroscopically defined dynamics**

For small values of the time step parameter Δ*t* and step size parameter σ, Verdonck and Tuerlinckx (2014) provide a set of stochastic diffusion equations that approximate the dynamics of the decision process (see Formula 19 and Appendix C in their paper). These equations, similar in form to those for the diffusion decision model and its variants, incrementally update the decision state of the system in discrete time and space by approximating the gradient of the free-energy surface through which the model’s evidence state moves. At each time step, the “drift rate” of IDM is calculated based on the gradient of the free-energy surface at the current position of the system, and the system is updated based on this gradient combined with Wiener noise.

##### **A typical decision trial**

Before the onset of the stimulus, the system stays at the place where the free energy of the surface is low (i.e., the start point of evidence accumulation). Once the stimulus is on, the free energy surface of the model changes dramatically to a polarized landscape, which is shown in Fig. 1e. There are two locally stable spontaneous states where the decision state wants to reach because of their lower free energy, each of which corresponds to one of the two responses. A decision is made when the state of the system reaches one of the two detection boxes corresponding to the two possible choices. A non-decision time is added to this first-passage time to yield a predicted RT. The black wiggly line in Fig. 1e depicts the trajectory of the system and Fig. 1f shows an example of correct and incorrect RT distribution of the IDM.

##### Simulating data from the models

Before we go into details on the specific methods for each model, the following was true for all models. For a given set of data, a set of parameter values must be chosen. For any parameter that varied across simulations, its value was drawn from a uniform distribution with a prespecified range (given in Tables 1, 2 and 3). When simulating data from a given model and a particular set of parameter values, we simulated *1,000* two-alternative forced-choice decisions. This process yields two distributions of RTs, one for each response (when the proportion of choices for either response is not 1). Any data set in which the choice proportion of a response was 1 was excluded, because we do not expect any individuals to exhibit perfect performance in 1,000 trials. In a similar attempt to restrict our analysis to data sets that are like what is usually done in practice, any simulated RTs greater than 3 s were excluded before any fitting was performed. In the analyses in the main text, the proportion of valid trials above 95% for all simulated data sets.

**Table 1:** Parameter values of the Drift-Diffusion Model (DDM) used for simulation and model fitting

Parameter	Description	Simulation range	Fitting range
*v*	Drift Rate	0–4	0–6
*a*	Boundary Separation	0.5–2	0.2–2
*Ter*	Non-decision Time	0.2–0.5	0.1–1

#### DDM simulation

The DDM data were simulated using custom-made code. The DDM has three core parameters: drift rate (*v*), boundary separation (*a*), and non-decision time (*T*_*er*_). All core parameters were randomly generated from uniform distributions with certain ranges, and other parameters were fixed to the default values given in Table 1. We generated 100 sets of parameters from a uniform distribution in one simulation round. For each simulation round with one set of parameters, the data of 1,000 trials were generated from the model. If the decision was not made after 3 s, the state was recorded as unfinished and removed from subsequent analysis.

#### OUM simulation

The OUM data were also simulated using custom-made code. The OUM has four core parameters: drift rate (*v*), boundary separation (*a*), non-decision time (*T*_*er*_), and effective leakage or self-excitation (*k*). All core parameters were randomly generated from uniform distributions with certain ranges and other parameters were fixed to the default values given in Table 2. We generated 100 sets of parameters from a uniform distribution.

**Table 2:** Parameter values of the Ornstein-Uhlenbeck Model (OUM) used for simulation and model fitting

Parameter	Description	Simulation range	Fitting range
*v*	Drift Rate	0–4	0–6
*a*	Boundary Separation	0.5–2	0.2–2
*Ter*	Non-decision Time	0.2–0.5	0.1–1
*k*	Effective Inhibition	-5–5	-15–15

#### IDM simulation

The IDM data were also simulated using the custom-made code. We used the IDM designed for two-alternative forced-choice tasks, which has a number of parameters that are always fixed when fitting the model to data. The values of these fixed parameters were set at values given in Table 1 in Verdonck and Tuerlinckx (2014). The default parameters are chosen because they can give rise to a decision field that can generate common dynamics of decisional evidence in simple decision-making tasks. Other patterns of the decision field can be found in Verdonck and Tuerlinckx (2014), Fig. 6.

**Fig. 3:**
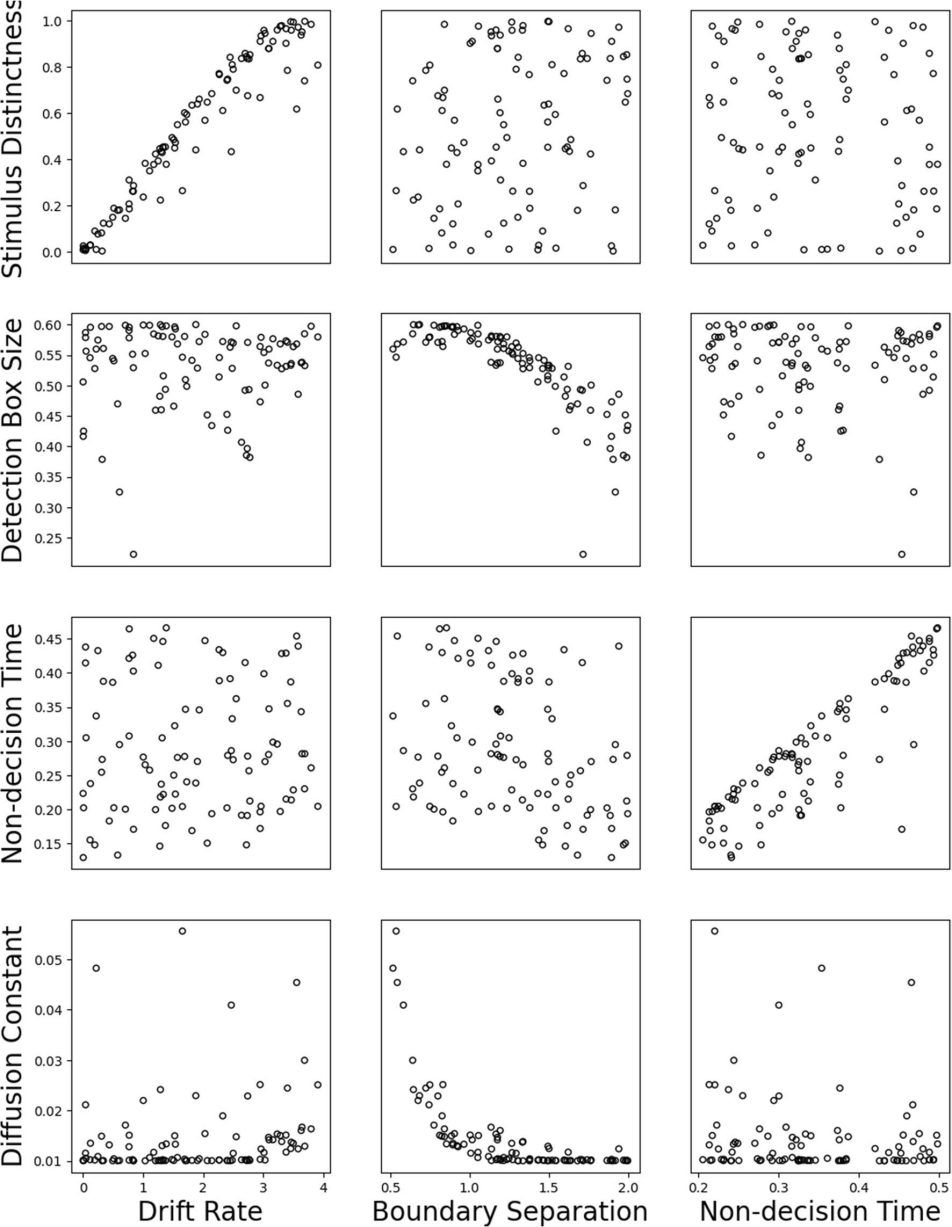
Results of fitting Ising Decision Maker (IDM) to Drift-Decision Model (DDM)  data. The horizontal axes show the core parameters in the DDM, and the vertical axes shows the analogous parameters in IDM. Subplots in the diagonal show the relationship between the corresponding parameters. Off-diagonal subplots show the relationship between other parameters

The IDM we used has six core parameters: stimulus distinctness (*C*), detection box size (*h*), non-decison time (*T*_*er*_), diffusion constant (*D*), self-excitation (*W*^*+*^), and mutual inhibition (*W*^*-*^). For the first cross-fitting analysis of IDM and DDM, only the first three varied and the other three were fixed according to the values given in Table 3. We initially hoped that the diffusion constant would be fixed in all cross fitting; however, we found that when fitting the IDM to DDM data, changing this parameter affects the identification of the detection box parameter (as shown in Online Supplementary Material (OSM) Figs. 2 and  3). Since we didn’t know what value to fix it to, we let the diffusion constant be free to vary parameter when fitting the IDM.

**Table 3:** Parameter values of the Ising Decision Maker (IDM) used for simulation and model fitting

Parameter	Description	Fixed value	Simulation	Fitting
*Θ*	Internal threshold	51450		
*N*	Total number of neurons	2000		
*β*	Inverse temperature	1/24		
*Δt*	Time step	1 ms		
*D*	Diffusion Constant	0.05	0.05	0.01-0.1
*B*_*ns*_	Baseline Strength of the External Field	2000		
*B*_*s*_	Input Strength	500	500	700
*C*	Stimulus Distinctness	0.5	0–1	0–1
*h*	Detection box size	0.4	0.2–0.6	0.2–0.6
*T*_*er*_	Non-decision time	0.4	0.2–0.5	0.1–1
*W* ^*+*^	Self-excitation	52500*	51000–52500	51000–52500
*W* ^*-*^	Mutual inhibition	8400*	8200–8600	8000–8800

Fixed value indicates same parameter values in simulation and fitting. Numbers written in the form a–b reflect ranges, and single values reflect values that were fixed (for either simulation or fitting)

*These values are fixed for the three- and four-parameter version of the IDM

For the second cross-fitting analysis of IDM and OUM, all the six core parameters are free to vary in data simulation. Since the self-excitation and mutual inhibition parameters of the IDM could relate to effective leakage parameter in the OUM, they were free to vary. For each simulation round with one set of parameters, the data of 1,000 trials were generated from the model. Every simulated trial started from the same starting point (0.2, 0.2), once the system crosses one of the detection boxes, the decision will be made if the detection boxes do not overlap at that point. If overlapped, the system will remain in the state of undecided and continue until only one of the detection boxes is crossed.

If the decision was not made after 3 s, the state was recorded as unfinished and removed from subsequent analysis. Finally, the mean activity of the two neural populations were clamped to the range of 0.001 and 0.999 for numerical stability.

### Model fitting

#### DDM and OUM fitting

To fit the DDM, we use the analytical method to obtain the likelihood of the full RT distribution using the Python package PyDDM (Shinn et al., 2020). We use differential evolution as the optimization algorithm, with robust BIC as the objective function. The robust BIC differs from BIC because it sets a minimal value (1e-20) for the likelihood to prevent infinite negative log likelihood (Shinn et al., 2020), which means if the likelihood is lower than this, it will be set to the minimum value. We estimated three DDM parameters: drift rate, boundary separation, and non-decision time. The ranges of the parameters we gave to the differential evolution optimizer are provided in Table 1.

To fit the OUM we use the approximation method implemented in the Python package PyDDM (Shinn et al., 2020). We again use differential evolution to optimize robust BIC to fit the model to data. We estimated four OU model parameters: input strength, boundary separation, non-decision time, and effective leakage. The ranges of these parameters used in the optimizer can be found in Table 2.

#### IDM fitting

We use the Mixed Neural Likelihood Estimation to fit the IDM to data (Boelts et al., 2022). This method uses a neural network to approximate the model likelihood, based on data simulated from the IDM. The training of this neural network has two steps: the aforementioned code is used to simulate training data sets that are used to train a conditional neural likelihood estimator that learns the relationship between the parameters of the IDM and the simulated data (i.e., RT and accuracy). After training, a joint density estimator can be used to give the likelihood of the model, so that parameter values can be inferred from empirical data using MCMC methods.

In the main body of the manuscript, we estimate four parameters of the IDM: stimulus distinctness (*C*), detection box size (*h)*, non-decision time (*T*_*er*_), and diffusion constant (*D*). The range of the parameters can be found in the Table 3. We chose to estimate *D* because fixing it led to much worse fits to data, as shown in OSM Fig. 6. The figure shows the detection box size of the IDM can only be mapped onto a small range (less than one-third of the specified range) of boundary separation in the DDM. Therefore, it is better to allow the diffusion constant free to vary as a scaling parameter. When fitting the IDM to DDM data, we present the results in which the leakage and inhibition parameters of the IDM are set at fixed values, but the results with all six parameters free to vary are also shown in OSM Fig. 7 (the same pattern emerges, albeit with more noise).

##### **Hyperparameters and training settings**

When training the neural network(s) that are used for fitting, we use 10^6^ simulated trials. Two neural networks are trained, both of which have of five layers and 200 neurons each. The neural network training was performed using the *sbi* package (Tejero-Cantero et al., 2020) with the following hyperparameters: learning rate 0.0005, training batch size 1000, 90%/10% training/validation split. The training converges if the validation loss does not improve after 20 epochs.

## Results

### Cross-fitting of IDM and DDM

The simulated data from one model was then fit with the other model to investigate the correspondence relationship between the two. First, since we are interested in the relationship between IDM and DDM, we fit DDM to the data generated by IDM and vice versa. The goal of this analysis is to determine whether the extra assumptions in the IDM influence the interpretation of the parameters that it shares with the simpler DDM. Second, we are also interested in the relationship between IDM and OUM, so fit OUM to data generated from IDM. Here, we are looking at the convergent validity of the parameters that are absent from the simplest evidence-accumulation models, like the DDM. Namely, do the OUM and IDM agree in terms of the interpretation of the leakage parameter in both models.

#### Fitting DDM to IDM data

**Fig. 4:**
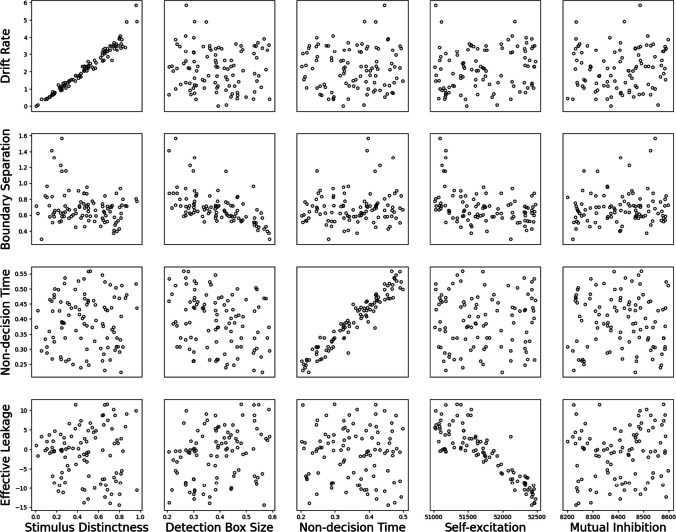
Results of fitting Ornstein-Uhlenbeck Model (OUM) to Ising Decision Maker (IDM) data. The horizontal axes show the core parameters in IDM and the vertical axes shows the analogous parameters in OUM. Subplots in the diagonal show the relationship between the corresponding parameters. Off-diagonal subplots show the relationship between other parameters

In the first part of our cross-fitting analysis, the DDM was fitted to data simulated from the IDM. Figure 4 shows the effect of changing the IDM parameters on the fitted DDM parameters. The three subplots on the diagonal of Fig. 4 show the relationship between analogous parameters of IDM and DDM, and the other subplots show the relationship between non-analogous parameters of both models.

From the left and the right columns of Fig. 4 we can see the changes in stimulus distinctness and non-decision time in IDM correspond exclusively to the changes in drift rate and non-decision time in DDM. For example, when the stimulus distinctness in IDM increases from 0 to 1, the estimated drift rate in DDM also linearly increases from 0 to 5, while the other DDM parameters (boundary separation and non-decision time) do not change. The effect of changing non-decision time was even simpler, such that changes in the IDM value have an almost identical mapping to the estimated non-decision time in DDM.

The middle column of Fig. 4 shows the effect of changing the detection box size in IDM on the three core parameters in DDM. When the detection box size in IDM changes from 0.2 to 0.6, the boundary separation parameter in DDM decreases from 0.4 to 0.3. Unlike the other parameters, however, the detection box size in the IDM has a nonlinear relationship with boundary separation in the DDM, with the relationship between the two parameters being much clearer for larger values of detection box size. Importantly, detection box size does not have any obvious effect on the other two DDM parameters.

Overall, we find that the core parameters of the DDM and IDM seem to correspond nicely. This result is encouraging, given that the DDM does not make the complex assumptions regarding the stimulus strength and the detection box in the IDM.

#### Fitting IDM to DDM data

We now fit the IDM to data simulated from the DDM. The pattern was similar to the previous result, but was noisier. Presumably, this is because the IDM has more complex assumptions and more parameters than the DDM, and so it is more difficult to identify its parameters (see OSM Figs. 3 and 5).

Figure 2 shows the relationship between the three core parameters in the DDM and three analogous parameters in the IDM (plus the diffusion coefficient). From the left column and the right column, we can see that the relationship between drift rate and non-decision time in the DDM and stimulus distinctness and non-decision time in IDM, respectively, is linear but noisy. Importantly, these parameters in the DDM do not seem to systematically affect the non-analogues parameters in the IDM.

As for the boundary separation parameter in DDM, from the middle column in Fig. 2 we can see that there is a relationship with the detection box size parameter in IDM, but not stimulus distinctness or non-decision time. Interestingly, the IDM’s diffusion constant parameter in the bottom row also decreases when the boundary separation parameter increases. That is, the diffusion coefficient seems to capture valid information from DDM’s boundary separation parameter. OSM Fig. 6 shows that if the diffusion coefficient is fixed, then the relationship between boundary separation and detection box size is much less clear.

To sum up, fitting the IDM to DDM data revealed a mostly similar pattern to what we saw in the preceding section. However, we again see that detection box size and boundary separation have the least clearly equivalent relationship. That said, the degree of overlap is impressive given the vastly more complex assumptions in IDM compared to the DDM.

### Cross-fitting of IDM and OUM

#### Fitting OUM to IDM data

Figure 4 plots the results of fitting OUM to data simulated from the IDM. Looking first at the “core” parameters of drift rate, boundary separation, and non-decision time, we see that there is a selective and linear relationship between stimulus distinctness in IDM and drift rate in OUM, as well as for non-decision time in both models. The relationship between detection box size in the IDM and boundary separation in the OUM is mostly linear, and it is reassuring that changes in box size do not clearly map onto any other parameter of the OUM.

In terms of the more “complex” assumptions, we also see a relationship between self-excitation in the IDM and leakage in the OUM. We see that for values of self-excitation above the internal threshold value of 51450 (see Table 3), the effective leakage parameter *k* in OUM is negative. This result makes sense, since self-excitation values above threshold (*W*^*+*^* > Θ*) and negative effective leakage (*k* < 0) both ambiguously correspond to the idea of self-excitation. It is also worth noting that for this part of the simulated data (and fits), the relationship between the IDM and OUM parameters is clear, linear, and strong (*r*(54) = -.89, *p* < .001, if we focus on only the data where *k* < 0). If we now look at values of self-excitation below threshold, we see that values of *k* are now positive, since both parameters now reflect effective leakage of activation. However, surprisingly, we see that the relationship between the IDM and OUM parameters practically disappears. For example, the correlation between the two parameters shrinks to -0.29 (*r*(46) = -.29, *p* = .048) if we look at simulations where k > 0, and what relationship there is likely carried by one unusual data point. Indeed, if we instead split the data based on whether the self-excitation parameter is above or below the internal threshold, then the correlation between parameters is only -0.01 (r(39) = -.01, p = .951) when we focus on the “leakage” simulations (i.e., where *W*^*+*^* < Θ* ).

To sum up, we find that the OUM leakage parameter corresponds to the IDM self-excitation parameter only when it reflects a self-excitation process. We return to the implications of this for cognitive psychometric applications of the OUM in the *Discussion* below. Finally, we see no noticeable relationship between mutual inhibition in the IDM and any of the parameters in the OUM.

In summary, the corresponding parameters related to stimulus strength, non-decision time, and self-excitation/leakage have linear relationships between IDM and OUM. Interestingly, the relationship between boundary separation and detection box size appeared to be much clearer for the OUM and IDM than it was for the DDM. One possible reason could be that the nonlinear relationship between detection box size in the IDM and boundary separation in the DDM was due to the influence of the mutual inhibition and self-excitation parameters in the IDM. Because the OUM could account for any effect of these parameters with its own leakage (self-excitation) parameter, it seemed more selectively influenced by detection box size.

It was perhaps surprising to see no influence of the mutual inhibition parameter on any of the parameters of the OUM. However, we found in parameter recovery studies that the IDM was unable to recover its mutual inhibition parameter (see OSM Fig. 4). As such, it may not be a property of the role of mutual inhibition in the IDM theoretical framework, but more that this parameter has no systematic influence on RT and accuracy rates.

Finally, it is worth noting that OSM Fig. 8 shows the fit of IDM to OUM data. The IDM has six free parameters, and so the parameters are estimated rather noisily. We see a similar pattern of results that we saw when fitting IDM to DDM data, including the fact that both the diffusion coefficient and detection box size are influenced by changes in boundary separation. We also see those changes in the leakage parameter of the OUM is captured by changes in the self-excitation parameter of the IDM, but also by the diffusion coefficient. However, we again emphasize that the relationships between parameters are rather noisy, and we encourage that any attempt to use IDM in a cognitive psychometric exercise be accompanied by rigorous parameter recovery studies.

## Discussion

This study provides preliminary evidence that the simplifying assumptions of models like the DDM and OUM do not compromise their ability to estimate their core parameters. We see the parameters in IDM that share interpretations with those in the DDM also have a similar impact on their predictions for data. More specifically, the drift rate and non-decision-time parameters in the DDM can be readily interpreted like stimulus distinctiveness and non-decision-time in the IDM. The results are a little less clear for boundary separation in the DDM and detection box size in the IDM. Though detection box size has no obvious relationship with the other DDM parameters, it has a non-linear relationship with boundary separation, and suggests that perhaps the latter is a less sensitive measure to changes in caution (for want of a better term).

Interestingly, we found that the correspondence between OUM and IDM parameters was slightly better than between the DDM and IDM parameters. We see that the boundary separation parameter in the OUM has a more obviously linear relationship with detection box size in the IDM, and thus seems to offer a more sensitive measure of caution than in the DDM. One interesting conclusion of these results seems to be, therefore, that the OUM could be a better cognitive psychometric tool than the DDM – at least if we assume that more neurally plausible models such as the IDM are also more accurate models of decision-making.

Furthermore, we also see that the more complex assumption of self-excitation also shares a kind of “convergent validity” across the OUM and IDM, with the effective leakage parameter of the OUM and self-excitation parameter of the IDM having a similar influence on choices and RTs (indeed, this is foreshadowed in Figs. 1b, 1d, and 1f, comparing the shapes of RT distributions across the three models). This correspondence between self-excitation and effective leakage may have an implication for how we interpret this parameter. Throughout, we have referred to the *k* parameter in the OUM as effective leakage, in line with its typical role as a parameter that reduces the effective drift rate, so that the RT distribution will have a longer tail (Busemeyer & Townsend, 1993). We have shown, however, that the effective leakage parameter in OUM has better correspondence to the self-excitation in IDM when the “leakage” parameter is negative, resulting in a “self-excitation” effect. This means that instead of a longer tail, OUM with self-excitation can accelerate the evidence accumulation, leading to an RT distribution with a shorter tail. Our results suggest a revised interpretation of the OUM’s leakage parameter in cognitive psychometric applications of the model.

One might worry about the fact that we used different methods for fitting the IDM (MNLE) and the DDM and OUM models (likelihood-based methods). We could have used MNLE for the DDM and OUM, but this yields noisier parameter estimates than the likelihood-based method we were able to use, because DDM and OUM have known approximations for their likelihood functions. Since we would use the best available fitting method in any cognitive psychometric analysis, we thought it more important to reflect that aspect of reality than to control for fitting method and thus worsen our ability to measure DDM and OUM parameters. Similarly, it is likely possible to improve parameter estimation for the IDM by expending more computational resources. For example, perhaps mutual inhibition could be recovered better with a better trained neural network. However, it is important to note that in any evaluation of a model for use in Cognitive Psychometrics, the model and the fitting methods be evaluated together, since one of the reasons for using the simpler models in cognitive psychometrics is the fact that the more complex models require less efficient and less ideal fitting methods.

Our results also suggest that more work is needed to understand the impact of the more complex assumptions made by more neurally plausible decision-making models. For example, the mutual inhibition parameter in IDM is hard to recover, just like the self-excitation and inhibition parameters in the LCA (Miletić et al., 2017). Mutual inhibition can be recovered if it is the only parameter that varies, as shown in OSM Fig. 1. However, when both mutual inhibition and self-excitation are varied, then the ability to recover the parameter vanishes (see OSM Fig. 4), suggesting that self-excitation masks the effect of mutual inhibition. Further investigation is needed to disentangle the two theoretical concepts.

Finally, it is worth noting that the results here also only speak to just two of the ways in which decision-making models have become more neurally plausible (i.e., self-excitation and inhibition). Other work has looked at the relationship between other neurally plausible models, such as the spiking neural network model (Umakantha et al., 2022). Others still have proposed other theoretical mechanisms of decision making, such as urgency signals and collapsing bounds (Smith & Ratcliff, 2022). More work is needed to determine whether such factors can impact the appropriateness of the DDM or OUM as cognitive psychometric models.

In conclusion, this paper explores a critical issue in the realm of decision-making research, which is the balance between biological plausibility and practicality. The IDM, as a neurobiologically inspired model, aims for greater biological plausibility but as a result faces pragmatic computational challenges. The OUM is offered as a compromise between biological plausibility and practicality, seeming to offer a valid cognitive psychometric tool while also capturing one of the more neurally plausible assumptions. That said, the equivalence between the parameters of the DDM and the IDM is rather impressive given their relatively large differences in theoretical assumptions.

## Supplementary Information

Below is the link to the electronic supplementary material.

Supplementary file1 (DOCX 2.75 MB)
