# Supplementary material for: The neural implausibility of the diffusion decision model doesn’t matter for cognitive psychometrics, but the Ornstein-Uhlenbeck model is better
Source: Psychon Bull Rev. 2024 May 14;31(6):2724–36. doi: 10.3758/s13423-024-02520-5 (PMC11680627; doi:10.3758/s13423-024-02520-5)

# Appendix

## Parameter Recovery

### Parameter recovery of IDM (only mutual inhibition)

The parameter recovery result looks good for mutual inhibition if it is the only parameter to recover.

**Supplementary Figure 1**

*Parameter recovery of mutual inhibition in IDM*


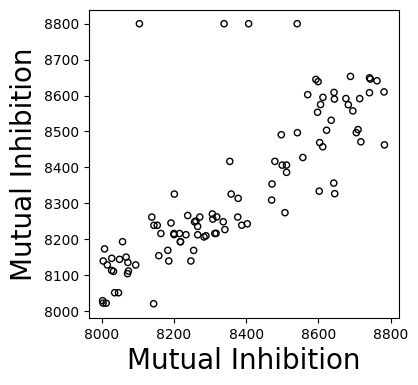


###

### Parameter recovery of IDM (self-excitation and mutual inhibition)

However, just adding one more parameter makes mutual inhibition impossible to recover.

**Supplementary Figure 2**

*Parameter recovery of self-excitation and mutual inhibition in IDM.*


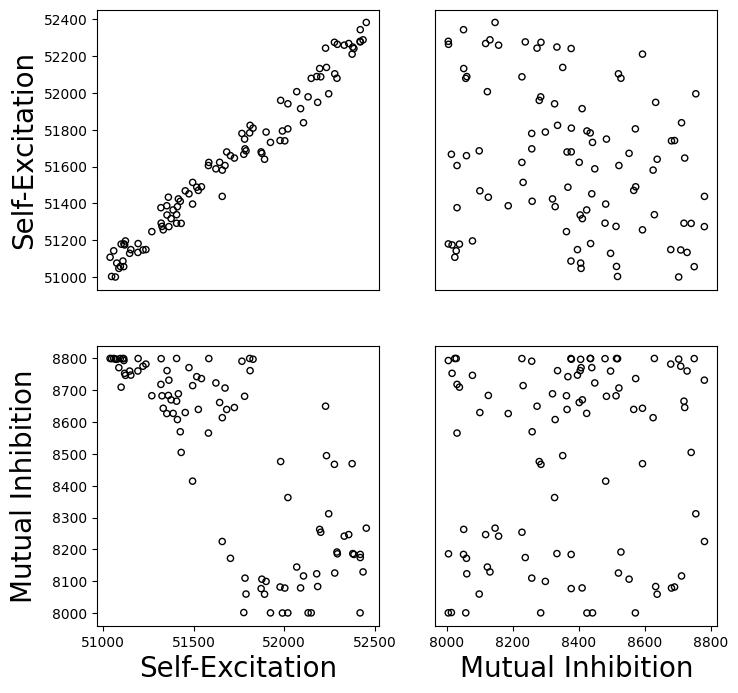


### Parameter recovery of IDM (three parameters)

**Supplementary Figure 3**

*Parameter recovery of stimulus distinctness, detection box size, and non-decision time*


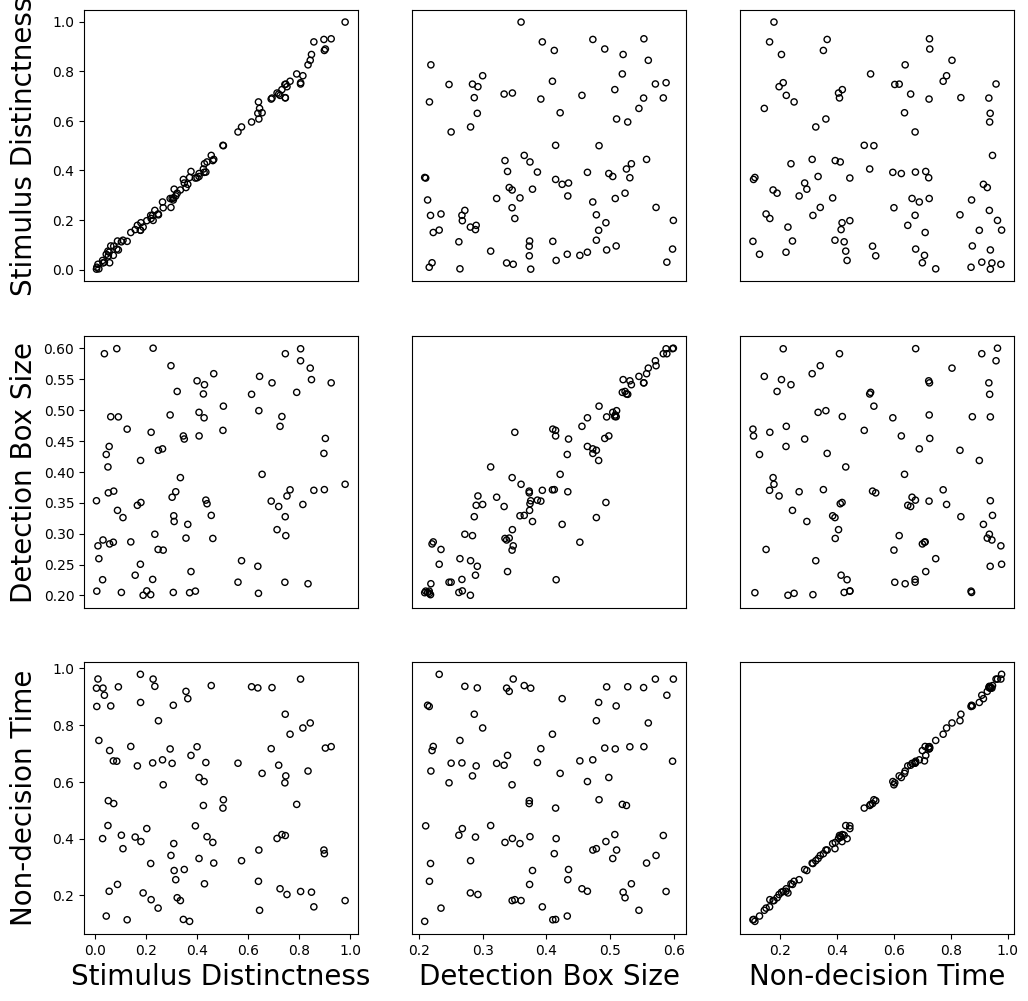


### Parameter recovery of IDM (four parameters)

**Supplementary Figure 4**

*Parameter recovery of stimulus distinctness, detection box size, non-decision time, and diffusion constant in IDM*


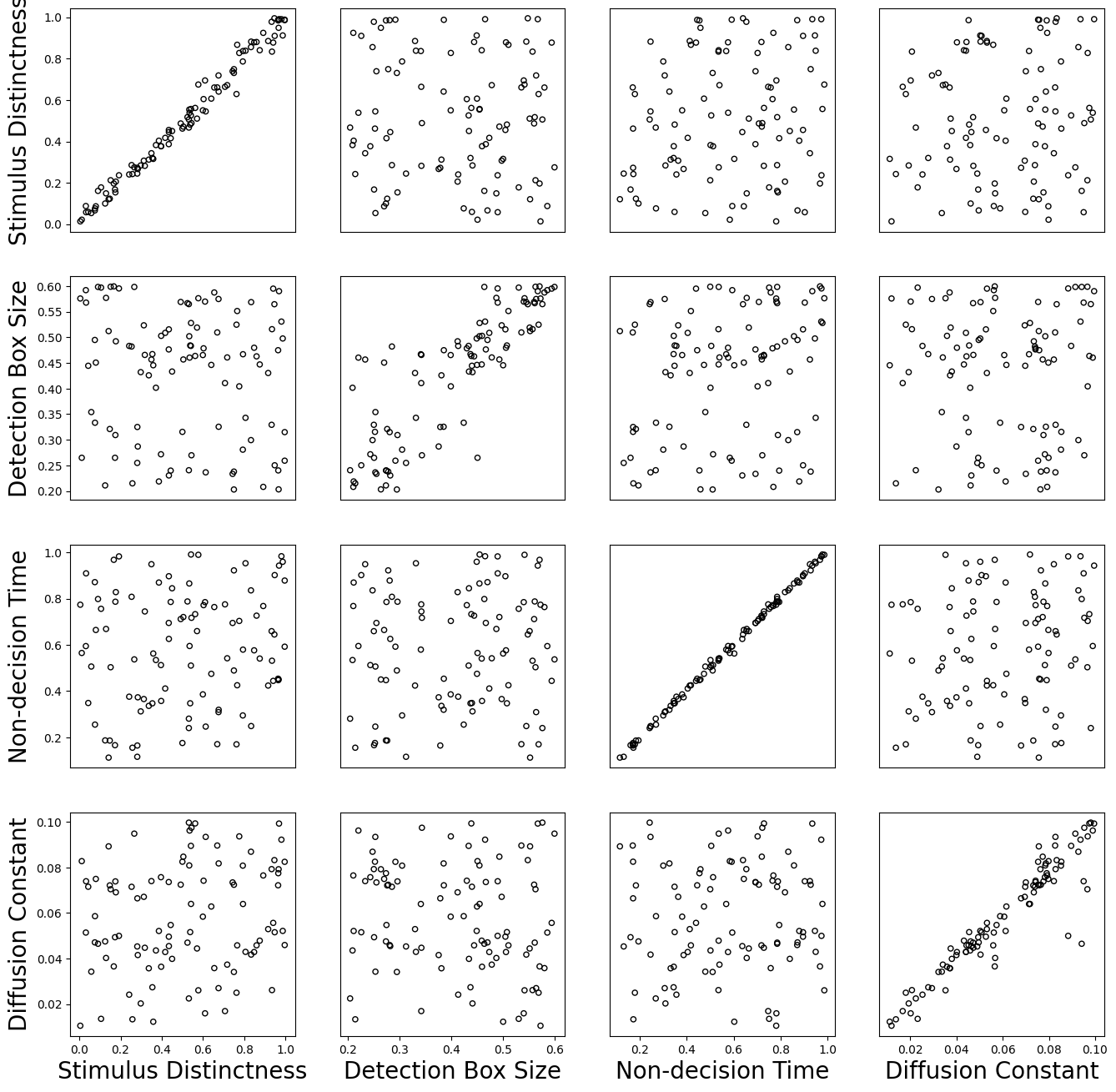


### Parameter recovery of IDM (all six parameters)

**Supplementary Figure 5**

*Parameter recovery of stimulus distinctness, detection box size, non-decision time, diffusion constant, self-excitation, and mutual inhibition in IDM*


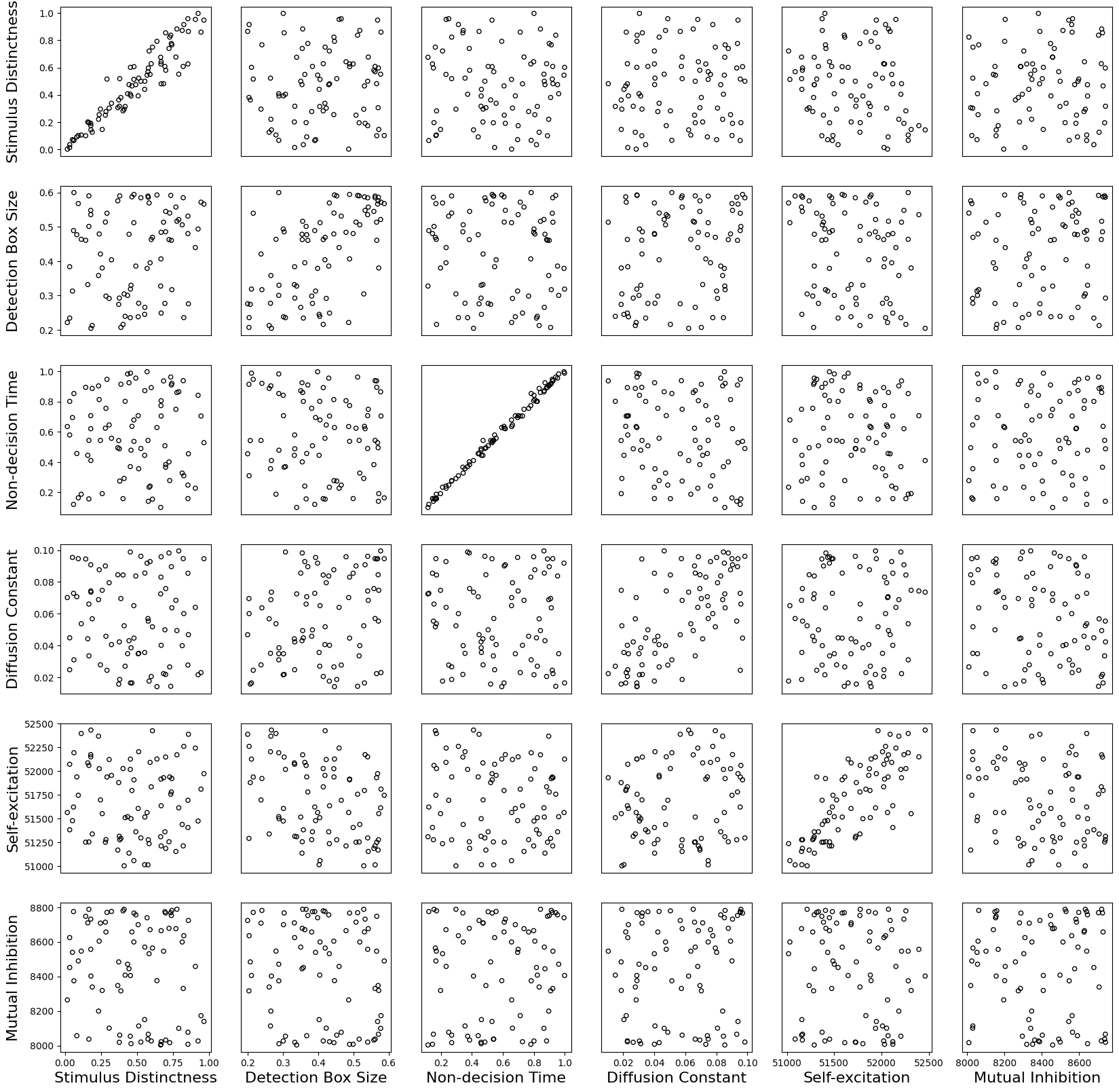


## Alternative Model Fitting

### Fit IDM (three parameters) to DDM data

**Supplementary Figure 6**

*Fit IDM (three parameters) to DDM data*


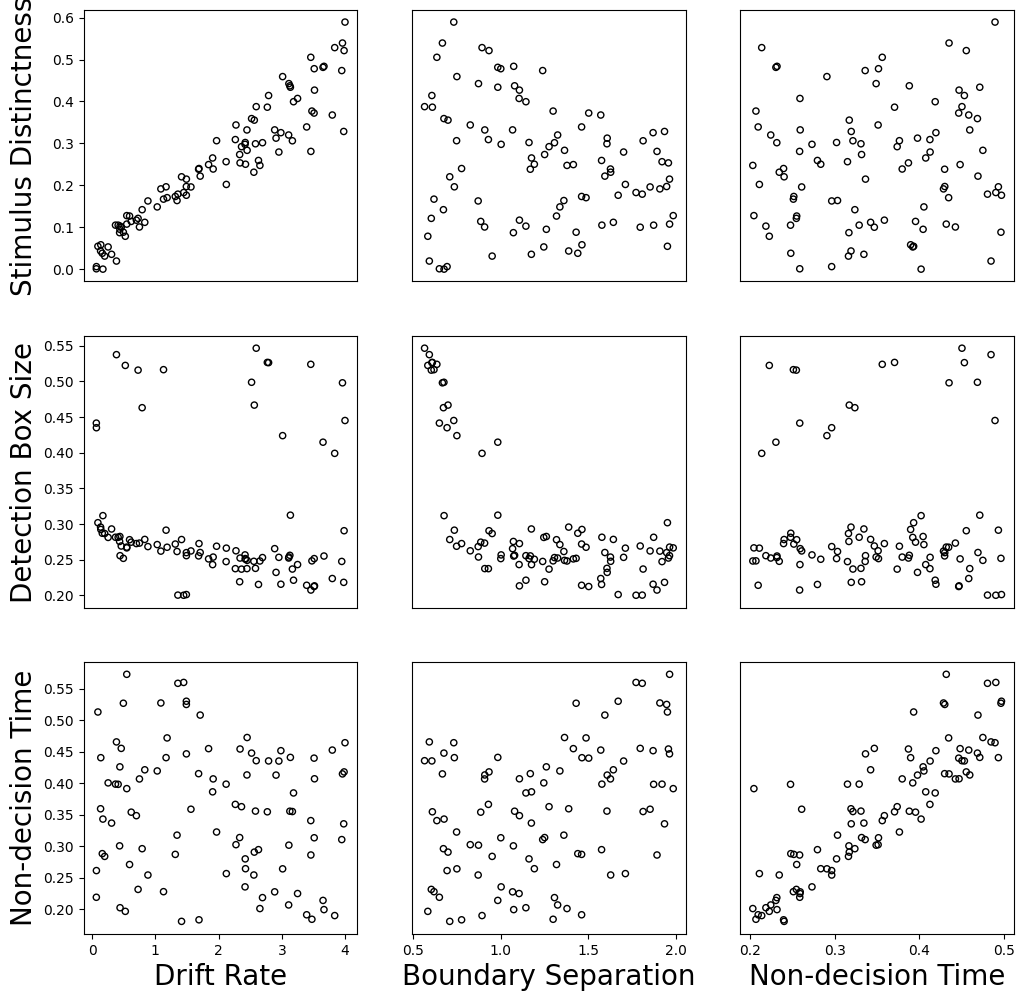


### Fit full IDM to DDM data

**Supplementary Figure 7**

*Fit full IDM (six parameters) to DDM data*


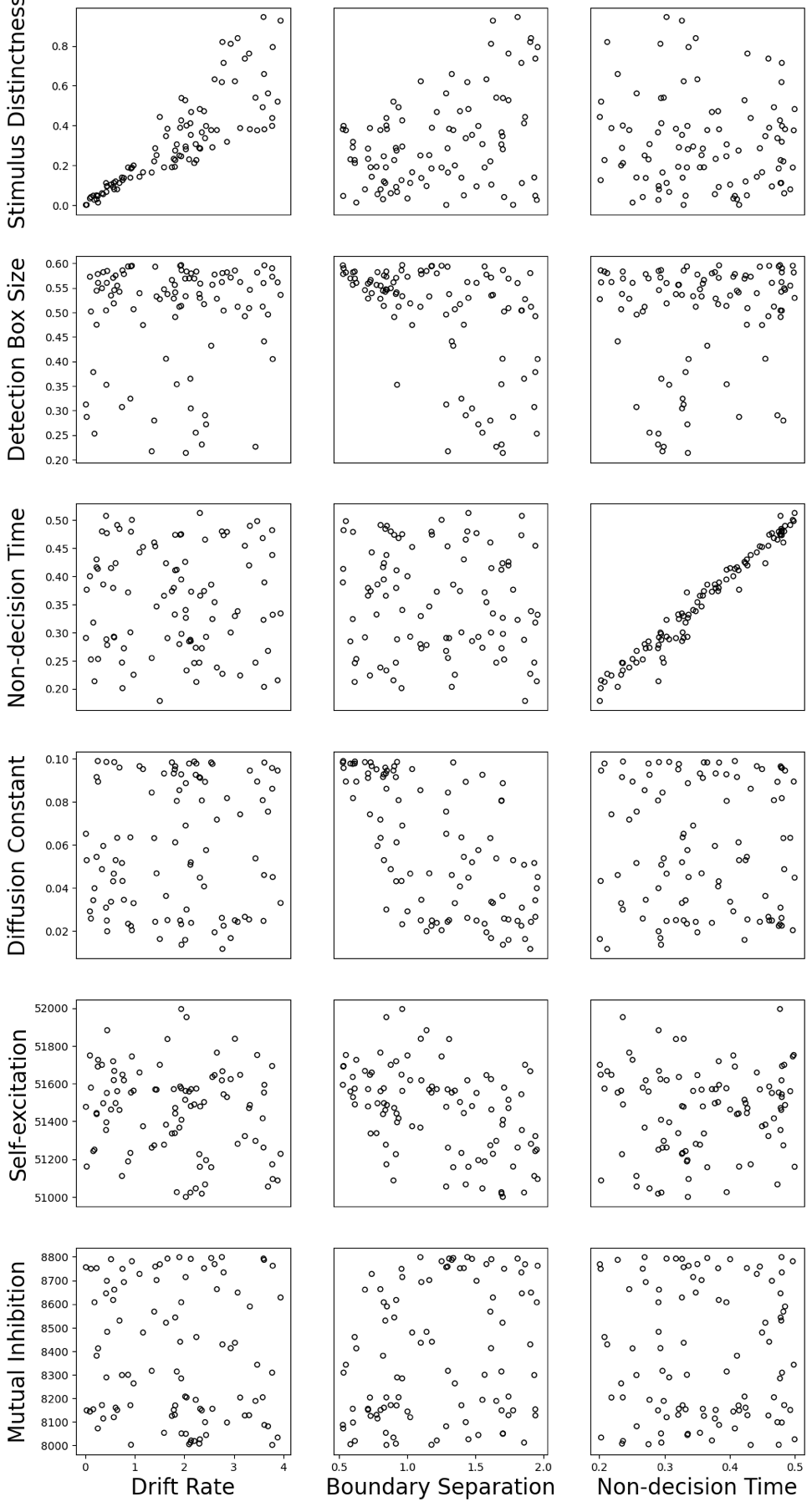


### Fit full IDM to DDM data

### Supplementary Figure 8

### Fit full IDM (six parameters) to OUM data


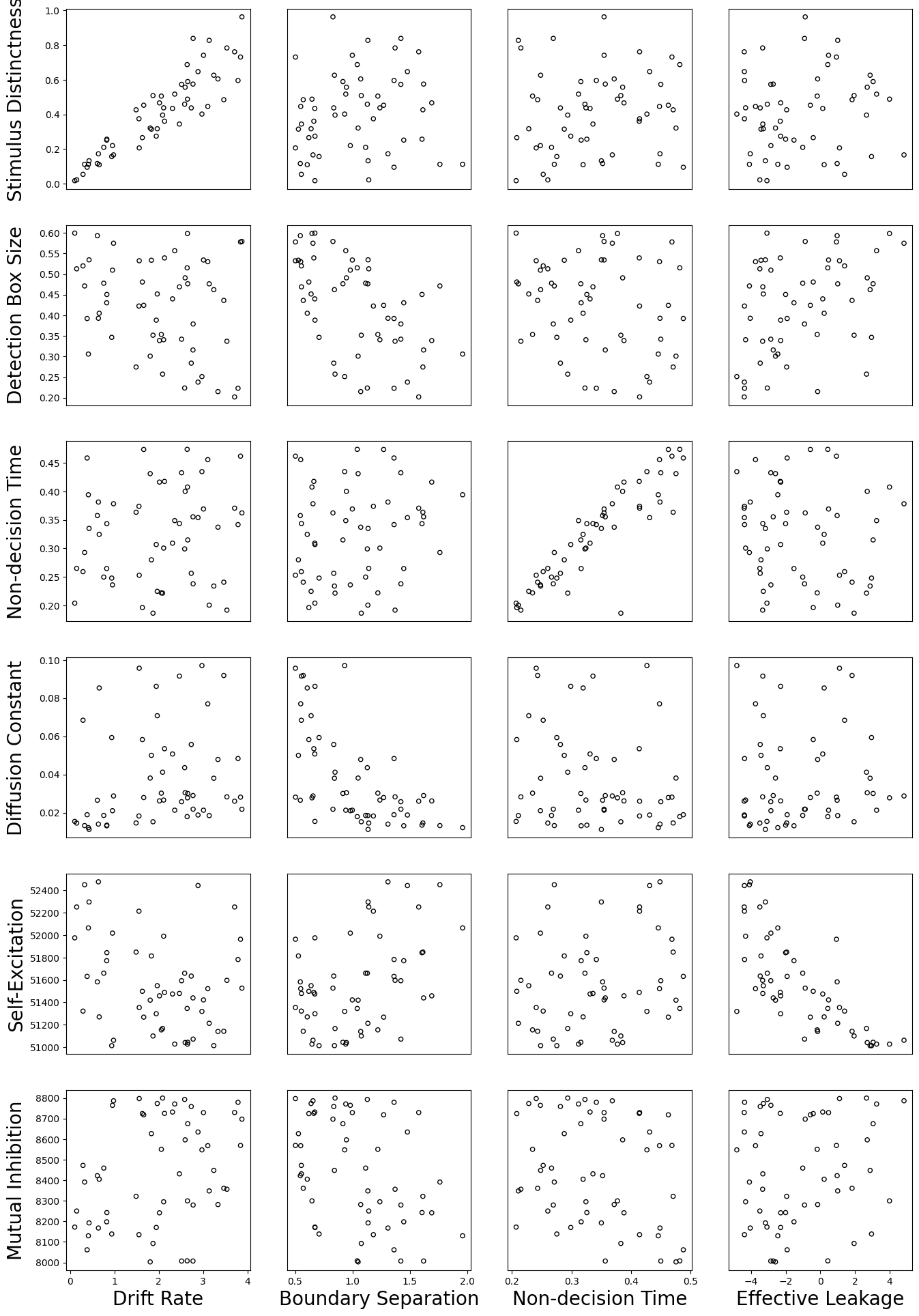


## Alternative shape of the Detection areas in IDM

### Right Triangle with legs h

**Supplementary Figure 9**

*Fit DDM to IDM (right-triangle detection area) data*


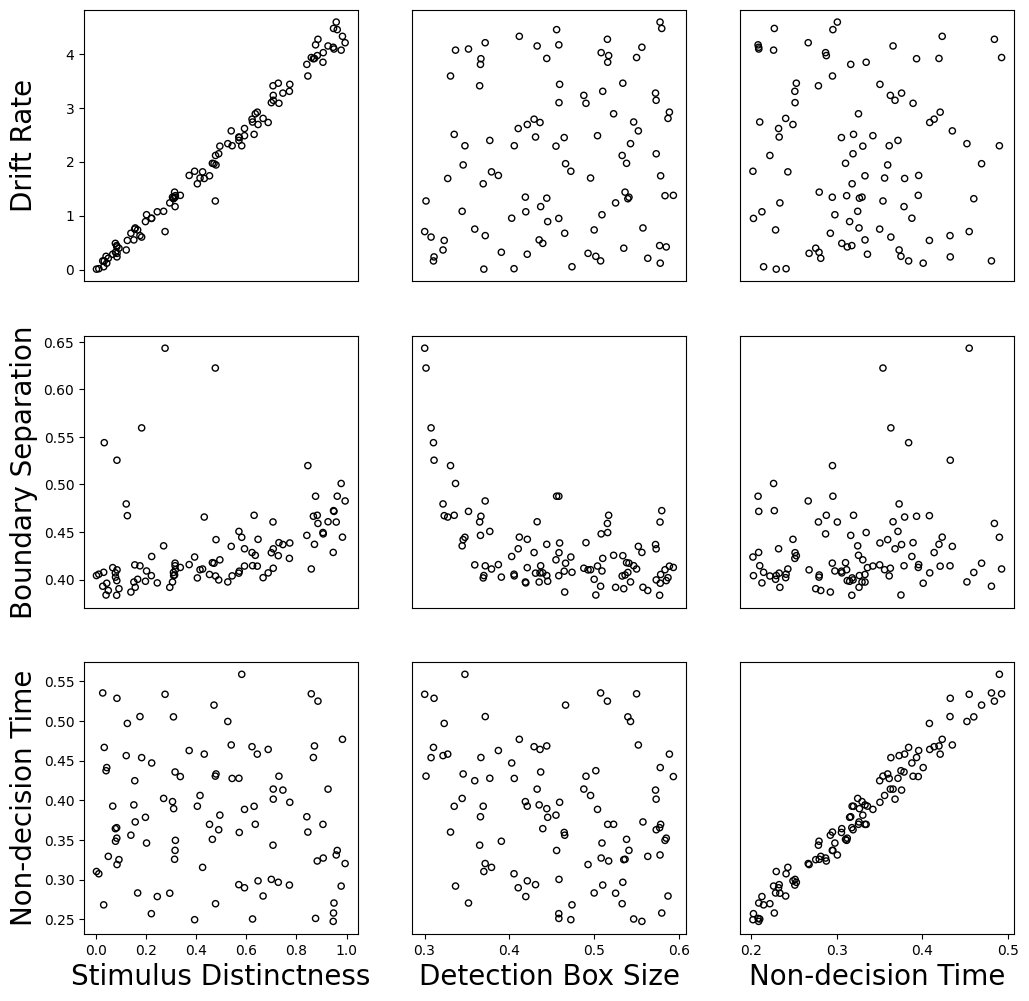


### Wedge with radius h

**Supplementary Figure 10**

*Fit DDM to IDM (wedge detection area) data*


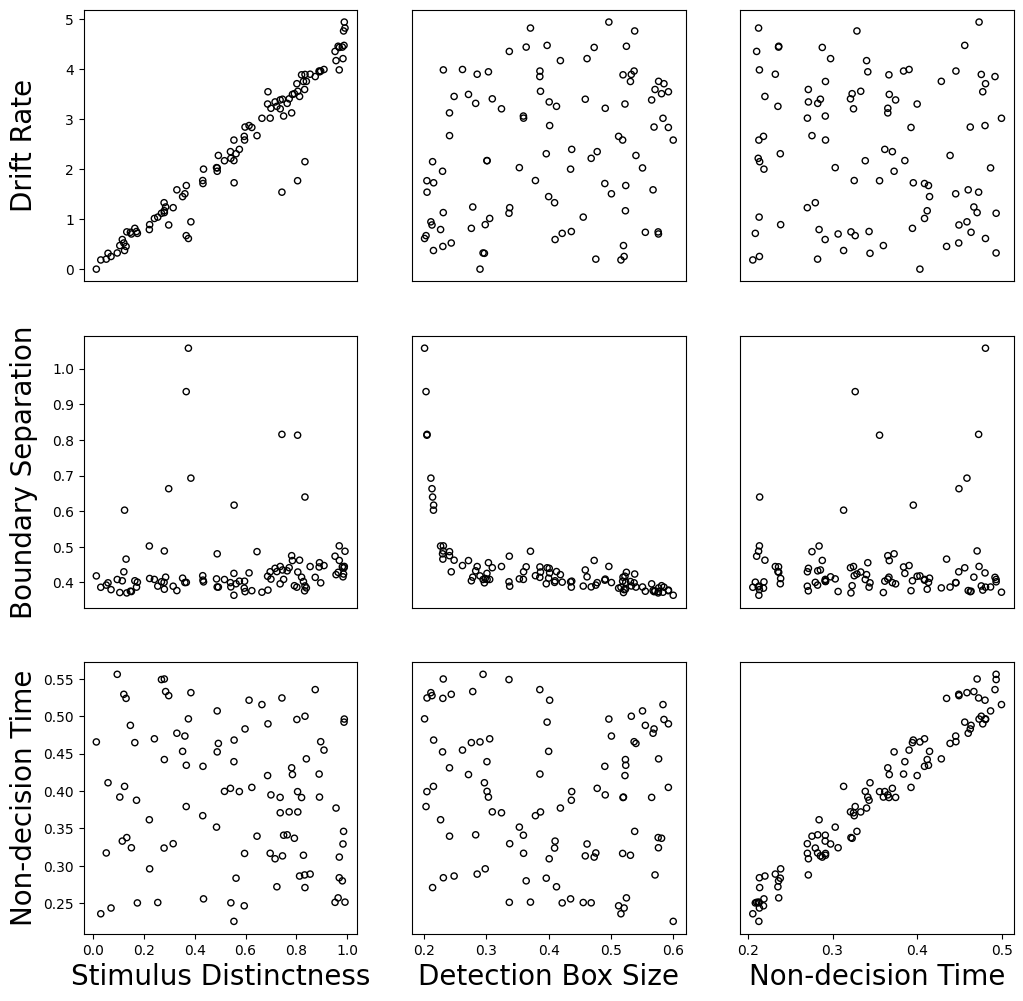

Supplement: Supplementary file 1 — Supplementary file1 (DOCX 2.75 MB) [file 13423_2024_2520_MOESM1_ESM.docx]
